# Thoracentesis

**Published:** 1861-01

**Authors:** 


### SELECTIONS.

*Thoracentesis.*—As we progress in the practical stndy of thoracentesis, we are more and more powerfully impressed with its innocuousness and the facility of its performance. We are now aware that it may prove beneficial even in the case of pleurisy consequent upon tuberculosis. Mr. Aran's general practice is to tap the chest, whenever the effusion is copious or increases with rapidity. In the latter case, he marks upon the skin with nitrate of silver the limits occupied by the morbid secretion, and watches its subsequent progress. If he finds after two or three days that its advance has been considerable, he unhesitatingly taps the chest, even when it is not entirely filled by the liquid, because further temporization might expose the patient to sudden suffocation. Again, when the effusion even moderate in quantity, does not yield to diuretics and repeated blistering, Mr. Aran has recourse to the same method. When the usual treatment has been unavailingly persevered in for eight days or a fortnight, he performs thoracentesis, and a cure is effected with surprising rapidity. Thus the three indications for thoracentesis are : Considerable effusion ; rapidly increasing effusion ; obstinate effusion, whatever its amount.

Mr. Aran, moreover, cares little about the nature of the morbid secretion. Of course he would be glad to ascertain if it is or is not puriform, but, unable to acquire this knowledge, he takes the most favorable view of the circumstances, irrespectively of the complication, because the wisest conduct to be adopted is to remove a cause of inflammation and thus simplify matters.

One complication, however, gangrene of the lung, appears to Mr. Aran formally to forbid tapping the chest. It is not indispensable to select the most dependent part of the pleura for the puncture. The thoracic cavity is not an inert vase ; the liquid escapes with equal ease, whether the artificial aperture be high or low. The rule to be observed is to puncture the chest in that part in which the lung is most distant from the parietes.—(*Championniere's Journal.*)

